# Potential targets and applications of nanodrug targeting myeloid cells in osteosarcoma for the enhancement of immunotherapy

**DOI:** 10.3389/fphar.2023.1271321

**Published:** 2023-09-21

**Authors:** Jianshu Zhu, Jiawei Fan, Yuanliang Xia, Hengyi Wang, Yuehong Li, Zijia Feng, Changfeng Fu

**Affiliations:** ^1^ Department of Spine Surgery, The First Hospital of Jilin University, Changchun, China; ^2^ Department of Gastroenterology, The First Hospital of Jilin University, Changchun, China; ^3^ Department of Cardiac Surgery, The First Hospital of Jilin University, Changchun, China

**Keywords:** nanomedicine systems, myeloid cells, immunotherapy, tumor immune microenvironment, osteosarcoma

## Abstract

Targeted immunotherapies have emerged as a transformative approach in cancer treatment, offering enhanced specificity to tumor cells, and minimizing damage to healthy tissues. The targeted treatment of the tumor immune system has become clinically applicable, demonstrating significant anti-tumor activity in both early and late-stage malignancies, subsequently enhancing long-term survival rates. The most frequent and significant targeted therapies for the tumor immune system are executed through the utilization of checkpoint inhibitor antibodies and chimeric antigen receptor T cell treatment. However, when using immunotherapeutic drugs or combined treatments for solid tumors like osteosarcoma, challenges arise due to limited efficacy or the induction of severe cytotoxicity. Utilizing nanoparticle drug delivery systems to target tumor-associated macrophages and bone marrow-derived suppressor cells is a promising and attractive immunotherapeutic approach. This is because these bone marrow cells often exert immunosuppressive effects in the tumor microenvironment, promoting tumor progression, metastasis, and the development of drug resistance. Moreover, given the propensity of myeloid cells to engulf nanoparticles and microparticles, they are logical therapeutic targets. Therefore, we have discussed the mechanisms of nanomedicine-based enhancement of immune therapy through targeting myeloid cells in osteosarcoma, and how the related therapeutic strategies well adapt to immunotherapy from perspectives such as promoting immunogenic cell death with nanoparticles, regulating the proportion of various cellular subgroups in tumor-associated macrophages, interaction with myeloid cell receptor ligands, activating immunostimulatory signaling pathways, altering myeloid cell epigenetics, and modulating the intensity of immunostimulation. We also explored the clinical implementations of immunotherapy grounded on nanomedicine.

## 1 Introduction

Osteosarcoma (OS) is a specific type of malignant bone tumor that is particularly concerning in the medical community because it often affects adolescents and young adults. Like other types of malignant tumors, one of the challenges in treating osteosarcoma is chemotherapy resistance. This resistance implies that conventional chemotherapy methods have limited effectiveness against osteosarcoma (Ritter and Bielack, 2010; Gill and Gorlick, 2021). Efforts are being made to identify specific molecular targets and promising innovative approaches in OS treatment (Chen et al., 2021; Wei and Yang, 2023). Osteosarcoma cells genetically differ from their normal counterparts, and tumor-associated antigens (TAAs) often have poor immunogenicity due to immune editing (Meltzer and Helman, 2021). The tumor continuously interacts with the host immune system, ultimately escaping immune surveillance. The tumor microenvironment consists of a complex system made up of tumor cells and the surrounding cells, molecules, and extracellular matrix. Within this microenvironment, interactions between tumor cells and the immunosuppressive cells and matrix cells in the tumor microenvironment form a network of immunosuppressive pathways, simultaneously inhibiting the activation of immune defense (Kansara et al., 2014; Isakoff et al., 2015). Therefore, treatments targeting immune mechanisms, such as immunotherapies, are especially significant. Checkpoint blockade is an immunotherapeutic approach. Under normal circumstances, these immune checkpoints help protect the body’s normal cells from attack (Wei et al., 2017; Guan et al., 2022). However, cancer cells might exploit these immune checkpoints to evade surveillance by the immune system (Postow et al., 2018; Kalbasi and Ribas, 2020). Consequently, by inhibiting certain signals, the ability of immune cells to combat tumor cells is enhanced (Anwar et al., 2020). The key to successful immunotherapy is overcoming local immune suppression in the tumor microenvironment and activating mechanisms leading to tumor eradication (Li et al., 2023). Chimeric Antigen Receptor (CAR) T-cell therapy is another cutting-edge technique in which T cells are genetically engineered to recognize and attack “chimeric” cells carrying specific tumor antigens (Porter et al., 2011; June and Sadelain, 2018). Once these T cells are redirected and proliferated, they are reinfused into the patient, exerting their immunotherapeutic effects against cancer cells (Grupp et al., 2013; Yu et al., 2019). This method has demonstrated significant efficacy in the treatment of hematologic malignancies (Larson and Maus, 2021; Amini et al., 2022). However, so far, treatment of more common sarcomas such as osteosarcoma and solid epithelial cancers typically has not made a significant impact (Depil et al., 2020; Maalej et al., 2023).

But successfully activating myeloid cells to elicit anti-tumor immune responses poses several challenges, including 1) the heterogeneous nature of myeloid cell populations within the tumor microenvironment, 2) the potential for tumor-induced myeloid cell immunosuppression, and 3) the complexities associated with modulating myeloid cell functions without adversely affecting other crucial physiological processes. The intersection between nanomedicine and cancer immunotherapy is becoming the focal point of frontier developments in cancer treatment, with profound therapeutic prospects. Nanodrugs, as the core carrier of this interdisciplinary field, bring revolutionary possibilities for cancer immunotherapy (Lakshmanan et al., 2021). In cancer immunotherapy, the emergence of nanodrugs offers a more precise and targeted approach. Nanodrugs can effectively improve drug delivery, increasing its concentration in tumor cells while reducing toxicity to normal cells (Fang et al., 2018; Fang et al., 2023). An increasing number of preclinical and clinical data indicate that combining nanodrugs with immunotherapy can enhance therapeutic effects by activating immune responses in the tumor microenvironment (Duan et al., 2019a; Liu and Sun, 2021). When targeting cancer cells, nanodrugs generally aim to induce immunogenic cell death, triggering the release of tumor antigens and danger-associated molecular patterns, such as calreticulin translocation, high mobility group box 1 protein, and adenosine triphosphate, capable of inducing immune responses to eliminate tumor cells (Kepp et al., 2021; Fu et al., 2022). In this process, the release of high mobility group box 1 protein (HMGB1) plays a key role, as it can serve as an alarm signal, promoting antigen presentation processes and inducing a stronger immune response (Inoue and Tani, 2014; Stagg et al., 2023). Nanodrugs targeting the tumor immune microenvironment enhance cancer immunotherapy by inhibiting immune suppressive cells (such as M2-like tumor-associated macrophages) and reducing the expression of immune suppressive molecules (such as transforming growth factor β) (Binnemars-Postma et al., 2017; Baig et al., 2020) Moreover, myeloid cells, like M2-like tumor-associated macrophages, are essential components of the tumor immune microenvironment playing an immunosuppressive role, often expressing factors that inhibit immune responses, such as transforming growth factor β (TGF-β). Nanodrugs can target these immunosuppressive myeloid cells, thereby modulating the tumor immune microenvironment (van der Meel et al., 2013; Jin et al., 2018).

In this review, we discuss the mechanisms of preclinical model immunotherapies based on nanomedicine that enhance the therapeutic effect of the immune system by targeting myeloid cells. We focus on strategies established in nanomedicine that complement those established in genetic engineering and molecular biology, potential therapeutic targets, and applications of nanodrugs targeting myeloid cells, particularly tumor-associated macrophages, in osteosarcoma to strengthen immunotherapy. However, due to the heterogeneity of different tumors and individuals, nanoplatform delivery systems may not be effective for all types of tumors. Lastly, we provide our views on the anticipated challenges and future directions of nanomedicine in the era of immunotherapy.

## 2 Mechanism of action of nanomedicine targeting myeloid cells

**FIGURE 1:**
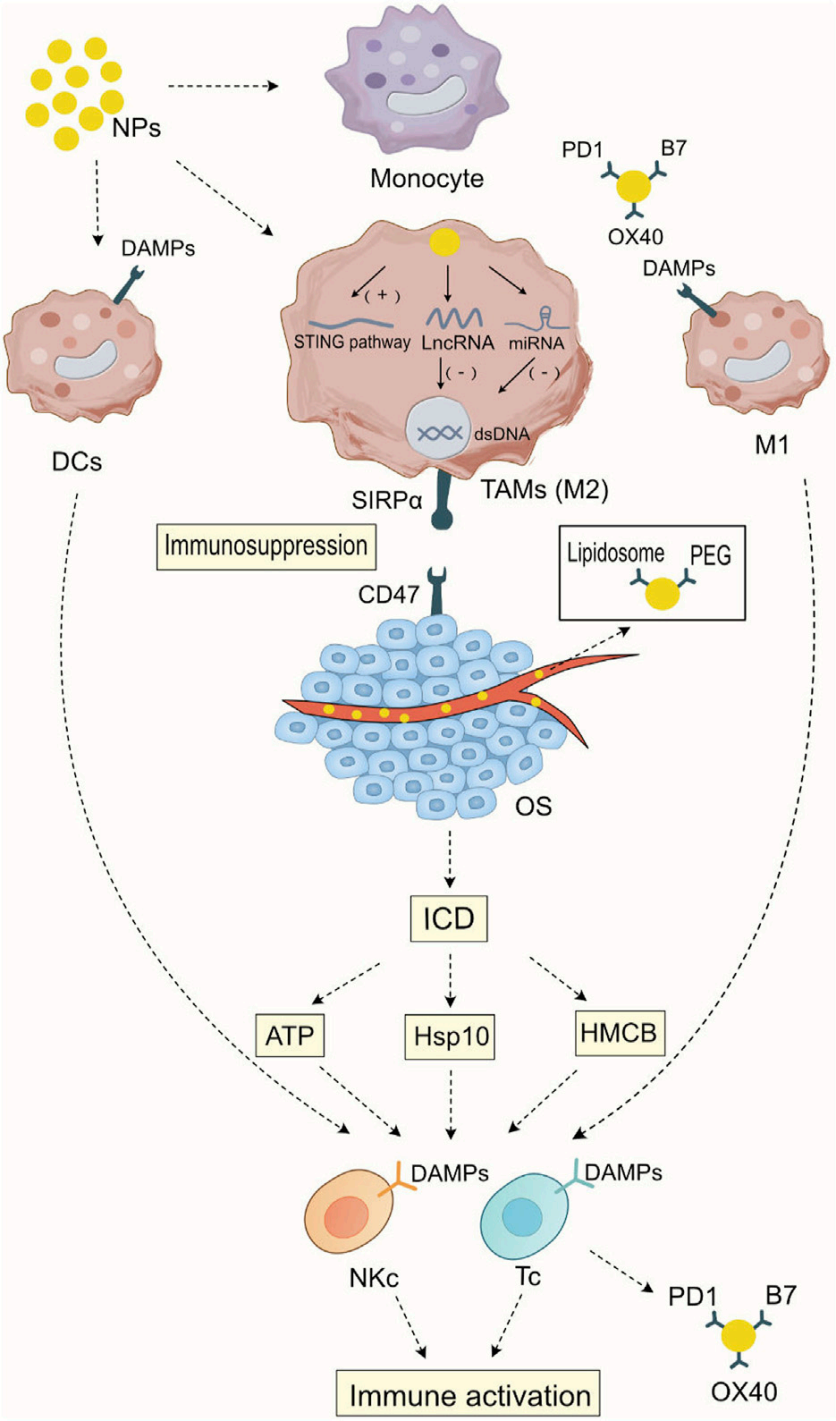
Mechanism of Action of Nanoparticles Targeting Bone Marrow Cells in the Modulation of Immunotherapy After being phagocytosed by myeloid cells, nanoparticles can activate the STING signaling pathway in tumor-associated macrophages, thereby initiating cellular immunity. At the same time, they can release the carried LncRNA or miRNA to silence specific gene expressions, leading to a transformation in the macrophage subtype. Nanoparticles carrying specific ligands such as PD1, B7, and OX40 can bind to specific ligands on dendritic cells and M1-type macrophages, enhancing the antigen presentation ability of dendritic cells and M1-type macrophages and strengthening the specific immunity against tumor cells. Nanoparticles carrying chemotherapy drugs have a direct cytotoxic effect on tumor tissues, promoting immunogenic cell death (ICD) and thus enhancing the direct cytotoxic effects of T cells and NK cells on tumor cells. In addition, during blood transport, nanoparticles wrap Lipidosomes and PEG to reduce cytotoxic side effects on normal tissues, enhancing the specificity of immunotherapy.

Nanomedicine provides new mechanisms of action for immunotherapy, including promoting immunogenic cell death, regulating the proportion of different cell subgroups in the myeloid cells, presenting immune-stimulating ligands to immune cells, activating immune-stimulating signal transduction pathways, delivering nucleic acids to cells, changing epigenetics, and controlling the intensity of immune stimulation (Yang et al., 2021) In this section, we briefly introduce the mechanisms of nanomedicines targeting myeloid cells and their potential role in osteosarcoma treatment. As illustrated in Figure 1, Mechanism of Action of Nanoparticles Targeting Bone Marrow Cells in the Modulation of Immunotherapy.

### 2.1 Abnormal phagocytic function of M1 macrophages and immune-inflammatory injury promote immunogenic cell death

**FIGURE 2:**
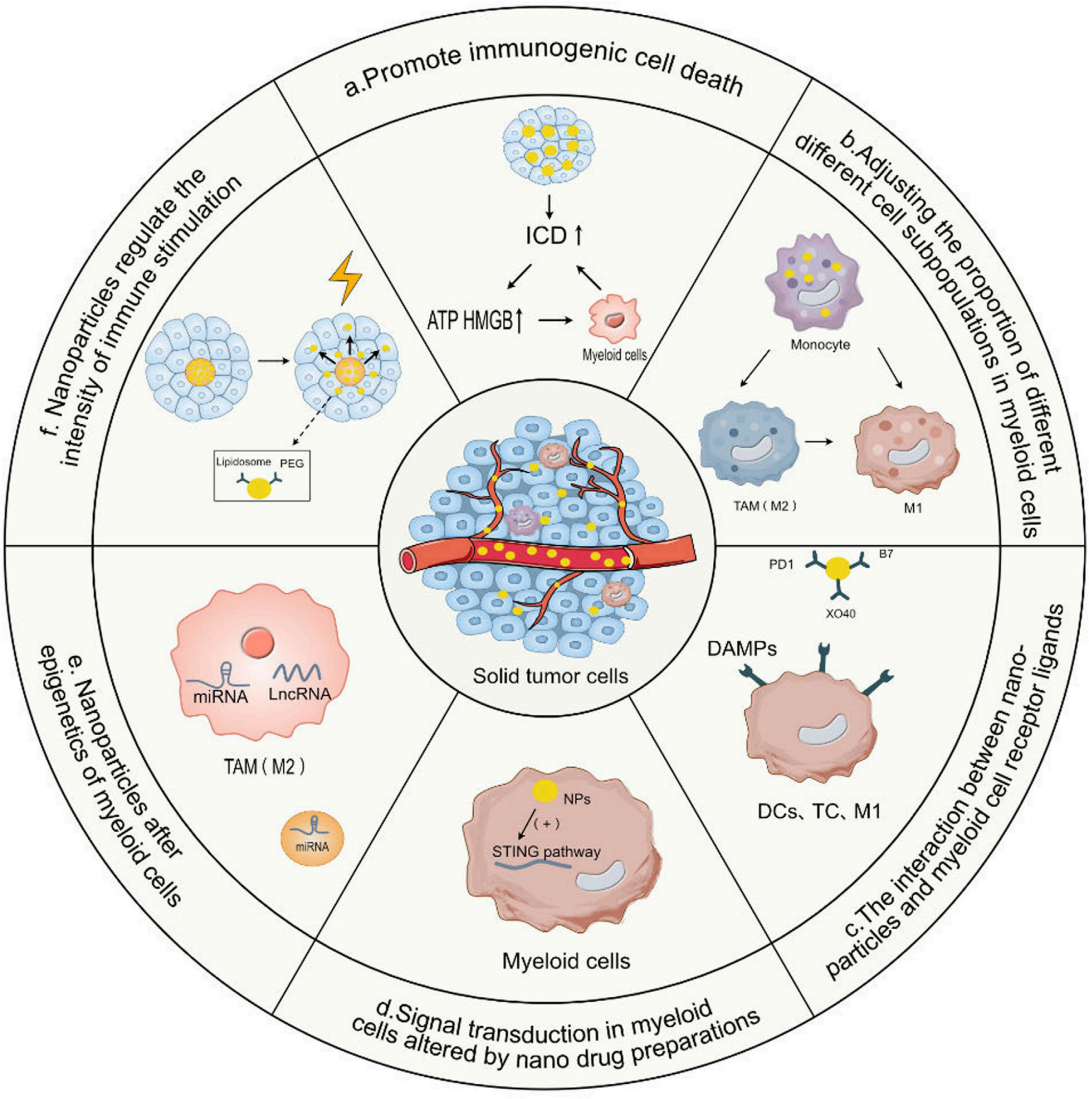
The mode of action of nanoparticles targeting myeloid cells in regulating immunotherapy. **(A)** Promote immunogenic cell death. Nanoparticles promote the release of ATP and High Mobility Group Box 1 (HMGB1) by promoting tumor immunogenic cell death (ICD), thereby activating the uptake of tumor antigens by antigen-presenting cells in myeloid cells and their subsequent activation, thereby enhancing the direct killing effect of T cells and NK cells on tumor cells. **(B)** Adjusting the proportion of different cell subpopulations in myeloid cells. By targeting myeloid cells with a nanodrug system and then adjusting the proportion of different subgroups of tumor-associated macrophages, the effects of T cell immunotherapy can be enhanced by depleting the M2 macrophage subpopulation and MDSCs in TAMs. **(C)** The interaction between nanoparticles and myeloid cell receptor ligands. Nanoparticles provide a series of ligands or release cytokines, co-stimulatory signals DAMPs provided by myeloid antigen-presenting cells to T cells and natural killer cells (NK), receptor-ligand interactions, activating dendritic cells (DCs), T cells and natural killer cells (NKC). **(D)** Signal transduction in myeloid cells altered by nano drug preparations. Nanodrug formulations can directly deliver drugs to myeloid cells, changing the signal transduction of myeloid cells and enhancing their anti-tumor activity. **(E)** Nanoparticles Alter Epigenetics of Myeloid Cells. Nanoparticles carrying miRNA are internalized in myeloid cells such as M2 TAMs and solid tumor cells, and siRNA is released, leading to effective gene silencing. **(F)** Nanoparticles regulate the intensity of immune stimulation. Nanodrug formulations can be designed to interact with external energy sources such as light or heat, thereby precisely controlling the timing of drug release and the intensity of myeloid cell immune stimulation, or controlling their bioactivity through liposomes, the delivery of prodrugs hidden by Polyethylene Glycol (PEG) chains.

The event of tumor cell death that encourages anti-tumor immune responses is known as Immunogenic Cell Death (ICD) (Yu et al., 2023). ICD is a unique form of cell death. ICD releases certain molecules, which in turn activate the immune system (Duan et al., 2019a). In the treatment of osteosarcoma, ICD is employed to activate the immune system, assisting the body in recognizing and eradicating osteosarcoma cells (Figure 2A).

ICD reveals that when tumor cells die, they release damage-associated molecular patterns (DAMPs), which in turn activate reactive immune cells, assisting the body’s immune system in recognizing and eliminating remaining cancer cells, such as ATP and high mobility group box 1 (HMGB1), as well as the surface exposure of calreticulin and heat shock protein 90 (HSP90) (Krysko et al., 2012; Ahmed and Tait, 2020). However, conventional cancer ablation treatments like chemotherapy or ionizing radiation exhibit different capacities to induce ICD, and their immunoenhancing effects may be counterbalanced by their toxicity to responsive immune cells (Adkins et al., 2014; Yan et al., 2020). Against this backdrop, nanodrugs promote the release of tumor antigens and DAMPs, allowing antigen-presenting cells to capture and present them to CD8^+^ T cells, leading to the activation of CD8^+^ T cells and enhancing their specific cytotoxic effects against cancer cells. Additionally, other myeloid cells, especially dendritic cells, also play a pivotal role during the ICD process. These cells can further enhance the activation and proliferation of T cells. Specifically, the increased expression of surface molecule CD80^+^ in myeloid cells further strengthens the immune response (Krysko et al., 2013).

Nanomaterial-induced ICD is also employed to boost combination therapies, either by co-encapsulating various drugs into the same particle to assure co-delivery to target cells or by inducing prominent ICD in tumors via combination treatment-loaded particles that result from the amalgamation of drugs with synergistic action patterns (Mishchenko et al., 2019). Additionally, emerging therapeutic approaches also harness external energy sources, such as light or heat, to interact with nanoparticles, thereby enhancing their therapeutic effects. For instance, nanoparticles can be designed to be sensitive to specific wavelengths of light or temperatures, synergistically producing ICD-inducing effects, thereby amplifying the exposure of calreticulin on the surface of tumor cells and the infiltration of immune cells (Guo et al., 2022).

### 2.2 Adjustment of the ratio of different cell subgroups within tumor-associated macrophages

By regulating strategies that transform TAMs from an M2 phenotype to an M1 phenotype, researchers aim to reshape the tumor microenvironment and promote anti-tumor immune responses. The latest developments in macrophage immunotherapy focus on strategies to reeducate TAMs from M2 to M1 phenotypes. Tumor-associated macrophages (TAMs) are defined as M2-type and are considered an important cellular subset in the tumor microenvironment that exerts immunosuppressive effects (Chen et al., 2019a). TAMs participate in various processes of tumor progression through the expression of cytokines, chemokines, growth factors, proteolytic enzymes, etc., thereby enhancing tumor cell proliferation, angiogenesis, and immune suppression, supporting invasion and metastasis (Pathria et al., 2019).

Utilizing the nanodrug system to target myeloid cells and then regulate the proportion of different subgroups of tumor-associated macrophages is a clinically attractive method because myeloid cells usually act as myeloid-derived suppressor cells (MDSCs) or TAMs with inhibitory effects, and they are reasonable therapeutic targets due to their tendency to phagocytize nanoparticles and microparticles (Zhu et al., 2020; Zhao et al., 2022). In mouse tumor models, intravenously administered nanoparticles easily accumulate in TAMs, and studies show that myeloid cells absorb ten times more nanoparticles than tumor cells (Kwong et al., 2013) Therefore, recent preclinical studies have tried to utilize this effect to eliminate the subgroup of myeloid cells that play an immune suppression role in the tumor immune microenvironment. Furthermore, M2 macrophages can inhibit CD8^+^ T cells to support tumor survival (Zhao et al., 2022). Therefore, T cell therapy can be enhanced by depleting MDSCs.

Another promising immunotherapeutic strategy to reduce inhibitory myeloid cells is to switch the phenotype that promotes anti-tumor immunity through reprogramming TAM (Li et al., 2021). The reversal of TAMs releases cytokines and gradually inhibits tumor angiogenesis, allowing for the remodeling of the tumor microenvironment (Figure 2B). Activated M1 macrophages are not only effector cells in innate immunity but also antigen-presenting cells that deliver processed antigens to T cells via MHC class II molecules, thereby promoting adaptive immune responses (Han et al., 2021). CD47^+^ is a protein expressed on the surface of cancer cells. It binds with the signal-regulatory protein α (SIRPα) on macrophages, producing a “don’t eat me” signal, which prevents the phagocytic activity of the macrophages. Blocking the CD47-SIRPα signaling axis and promoting repolarization from M2 to M1 in the tumor microenvironment can significantly prevent the local recurrence and distant metastasis of malignant tumors (Rao et al., 2020).

Furthermore, the latest findings in the field of immunometabolism indicate that macrophages, based on their polarization state, such as M1 macrophages mainly relying on glycolysis for energy and M2 macrophages primarily utilizing fatty acid oxidation and the TCA cycle for energy, display different metabolic characteristics among different macrophage subgroups (Ramesh et al., 2022). Therefore, these metabolic products are essential drivers of cellular signal transduction.

### 2.3 Interaction between nanoparticles and ligands of myeloid cell receptors

There are many key immune regulatory receptors involved in anti-tumor immunity, especially the co-stimulatory signals presented by myeloid antigen presenting cells to T cells and natural killer (NK) cells, involving cell-cell contacts and receptor-ligand interactions. The size and properties of nanoparticles enable them to serve as a carrier for antibodies and other therapeutic drugs, specifically delivering them to cancer cells (Irvine and Dane, 2020; Zhao et al., 2022). The interaction of nanoparticles with myeloid cell receptor ligands combines nanodrugs with immunotherapy, aiming to enhance the cancer immune response by enhancing key steps in the immune response cascade (Chaib et al., 2020).

The use of nanoparticles can optimize this process and enhance the immune response to cancer in several stages: nanoparticles can more specifically deliver drugs to cancer cells by loading ligands (such as antibodies or small molecules) onto nanoparticles, thus enhancing antigen release (Ifergan and Miller, 2020). Myeloid cells initiate immune responses by taking up and processing antigens. Nanoparticles can be designed to carry specific ligands or release specific signaling molecules that can stimulate myeloid cells, such as macrophages and dendritic cells, to more effectively take up and process antigens (Zhu et al., 2023). Myeloid cells play a pivotal role in T cell activation. This is achieved by presenting antigen fragments through their major histocompatibility complex (MHC) surfaces. Nanoparticles can be designed to enhance this process (Figure 2C), for example, by providing stimulating signals or directly presenting antigen fragments, DCs enhance tumor antigen presentation, leading to an increase in CD8^+^ T cell tumor infiltration, to more effectively activate T cells (Tuettenberg et al., 2016). Upon activation, the immune system’s key players, namely, Natural Killer (NK) cells and T cells, possess the capability to identify and subsequently eliminate cancer cells that express specific antigens. Nanoparticles carry specific ligands, and release signals that stimulate NK cells, and T cells, thereby enhancing their killing ability against cancer cells (Amoozgar and Goldberg, 2015).

In these processes, the interaction of nanoparticles with myeloid cell receptor ligands is crucial. Appropriate ligand design can make nanoparticles more targeted and more effectively activate the immune response. Overall, through these mechanisms, the combination of nanoparticles and immunotherapy can effectively enhance the immune response to cancer.

### 2.4 Activating the signal transduction pathway for immune stimulation

Nanodrug formulations can deliver drugs directly to myeloid cells, and nanodrug formulations play an important role in altering immune therapeutic drug-mediated myeloid cell signaling, thereby enhancing the anti-tumor activity of myeloid cells (Garner and de Visser, 2020). Nanodrug formulations can change immune therapeutic drug-mediated signal transduction, these drugs target cell signal transduction pathways in various ways to enhance their anti-tumor activity (Kumar et al., 2023).

Nanodrug formulations can provide multiple nanodrug formulations that can change immune therapeutic drug-mediated signal transduction (Figure 2D), these drugs target cell signal transduction pathways in various ways to enhance their anti-tumor activity (Darling et al., 2020; Xia et al., 2022). One of the main applications of nanomaterials in medicine is to promote intracellular drug delivery. Nanomaterials as alternatives to natural viruses have been widely studied to promote the entry of other drugs into the cytoplasm to change the signal transduction of myeloid cells (Fiering, 2017). Cancer immunonanodrugs mainly target TAM by blocking M2-type TAM survival or affecting its signal cascade, limit the recruitment of M2-type macrophages to tumors, and re-induce tumor-promoting M2-type TAM to anti-tumor M1-like phenotype, thereby enhancing the anti-tumor function of myeloid cells or inhibiting the immune-suppressive function of M2-type tumor-associated macrophages (Ovais et al., 2019). In addition, nanodrug formulations can be designed to have an impact on specific signaling pathways. For example, the key role of the stimulant of interferon genes (STING) pathway in anti-tumor immunity is by regulating the receptors or other signaling molecules on the surface of myeloid cells, thereby changing the signal transduction inside the cells, and further affecting the activity of myeloid cells (Luo et al., 2017). This is particularly important for manipulating the role of myeloid cells in immune responses.

### 2.5 Alteration of myeloid cell epigenetics by nanoparticles

Progress in the nanomedicine realm over the past years has laid the groundwork for the development of siRNA-based drugs as another category of personalized cancer immunonanodrugs (Zins and Abraham, 2020). Small interfering RNA (siRNA) therapies for cancer are increasingly becoming the focus of research interest (Huang et al., 2023).

Various microRNAs (miRNAs) disseminated by exosomes from tumors participate in intercellular communication (Hosseini et al., 2021). Nanoparticles often act as carriers for siRNA, similar to exosomes. siRNA primarily functions to reduce the expression of specific mRNA through the RNA interference (RNAi) mechanism, thereby inhibiting the production of corresponding proteins and altering the epigenetic state of myeloid cells (Xin et al., 2017; Kara et al., 2022). Furthermore, nanoparticles are readily internalized by phagocytic cells, enabling penetration into cells and potential interactions with biological macromolecules such as DNA and proteins (Ashrafizadeh et al., 2022). Nanoparticles carrying miRNAs exhibit high accumulation in myeloid cells and tumor tissues due to prolonged blood circulation and increased pH sensitivity. The current strategy involves both active and passive targeting, where nanoparticles carrying small interfering RNA (siRNA) are internalized into M2 TAMs (Tumor-Associated Macrophages) and solid tumor cells (Kanasty et al., 2013; Zhu and Palli, 2020) (Figure 2E). As the charge reversal occurs in the microenvironment where nanoparticles reside, nanoparticles exhibit effective endosome/lysosome escape and intracellular siRNA release, resulting in effective gene silencing (Song et al., 2018).

### 2.6 Regulation of the intensity of immune stimulation by nanoparticles

Nanomedicine can more precisely control the timing and location of immune stimulation, thereby maximizing therapeutic effects while reducing potential cellular toxicity.

Nanodrug formulations can be designed to interact with external energy sources such as light or heat, allowing for control over the timing of drug release and the intensity of myeloid cell immune stimulation (Kang et al., 2018; Chu et al., 2019). Furthermore, while combination therapies with anti-cell surface fusion antibodies have demonstrated significant initial anti-tumor activity, they have also resulted in lethal immunotoxicity caused by stimulating circulating white blood cells. To address this issue, researchers have proposed the use of liposomes as drug carriers. These tiny nanoscale vesicular structures can effectively anchor immunostimulants on their surface, ensuring rapid accumulation in tumor tissues while avoiding excessive exposure to the body as a whole (Zhang et al., 2018) (Figure 2F). Moreover, a third strategy in clinical development involves modifying immunostimulatory biologics with polyethylene glycol (PEG), converting them into inactive prodrugs. Once these prodrugs enter the body, they are activated, releasing their bioactivity, thereby ensuring the efficacy and safety of the treatment (Charych et al., 2016).

## 3 Preclinical and clinical research on nanodrugs targeting myeloid cells

Nanomedicine offers new opportunities and strategies for cancer treatment by integrating existing therapeutic methods with nanotechnology, aiming to provide safer and more effective treatment options. Here we enumerate the efforts made in preclinical and clinical research on targeted immunotherapy of myeloid cell cancer based on nanoparticles.

### 3.1 Treatment strategies for promoting immunogenic cell death

The ability to induce ICD varies among traditional cancer ablation therapies, and their immune enhancement effect can be counteracted by toxicity to responding immune cells (Duan et al., 2019a). Nanomedicine formulations present an appealing method for promoting ICD as they effectively induce ICD in cancer cells, which consequently enhances tumor immunogenicity, makes the tumor sensitive to anti-tumor T-cell immunity, and boosts the immunity of anti-tumor T-cells for cancer treatment (Guo et al., 2023).

Smart Nano Drug Delivery Systems (sNDDS) are at the forefront of nanoparticle technology. They allow targeted drug delivery and precise dosage control and amplify the immune response within the tumor by inducing ICD (Li et al., 2022). sNDDS combines the induction of ICD with cancer immunotherapy. For instance, synergistic effects are achieved by using ICD in conjunction with blocking the Programmed Cell Death Protein 1 Ligand and inhibiting the Indoleamine 2,3-dioxygenase 1 (Zhou et al., 2020). A phase I clinical study aimed at investigating the effects of the combined treatment of the IDO1 inhibitor navoximod with the PD-L1 inhibitor atezolizumab for advanced cancer indicated that 6 out of the dose-escalation group patients (9%) achieved partial clinical symptom relief. In the expansion group, 10 patients (11%) experienced either partial or complete clinical symptom relief (Jung et al., 2019). Therefore, although activity was observed, there is no definitive evidence to suggest a benefit of adding navoximod to atezolizumab.

Furthermore, the ability of nanomaterials to induce ICD is used to enhance the therapeutic effect of combination anti-tumor drug therapy, either by encapsulating multiple drugs in the same particle to ensure joint delivery to target cells or by combining the drugs with synergistic interaction modes to produce synergistically enhanced anti-tumor effects (Mishchenko et al., 2019; Yu et al., 2023). Platinum-based chemotherapy is widely used as a first-line treatment for a variety of cancers. PD-1/PD-L1 inhibitors have shown efficacy in a variety of cancers, and the combination of platinum-based chemotherapy and PD-1/PD-L1 inhibitors is gradually becoming a focus of attention (Liu et al., 2023; Ren et al., 2023). Recently, combination therapy has shown significant effects in preclinical models and clinical trials. For example, Oxaliplatin is derived from a metal coordinating group, allowing it to aggregate into solid particles in the presence of metal ions such as zinc. The combination therapy load particles generated by these nanoparticles induce significant ICD in tumors and synergize with anti-PD-L1 therapy in a mouse model (Duan et al., 2019b).

Nanoparticles are also designed to interact with external energy sources while carrying immunostimulatory drugs (Kobayashi and Choyke, 2019). In contrast to free photosensitizers 36, inorganic nanoparticles with a diameter of 25 nm coated with lipid-anchored photosensitizers enhance the exposure of calreticulin on the surface of tumor cells and infiltration of immune cells when combined with infrared light irradiation (Ji et al., 2022). This can cause photodynamic therapy and chemotherapy to produce an ICD inducing effect, leading to regressive changes in the tumor (Alzeibak et al., 2021; Guo et al., 2022). In addition, it has been reported that a laser/glutathione (GSH)-activated nanosystem has tumor penetration capabilities, allowing for efficient immunotherapy. Photodynamic therapy (PDT) is another crucial method for cancer treatment that kills cancer cells using a photosensitizer and light of a specific wavelength. OXA inhibits the growth of cancer cells and, in combination with PDT, induces ICD. Drug delivery activated by laser/glutathione is more advantageous for enhancing ICD and reversing the ITM in deep tumors. The chemotherapeutic PDTOPCPN@NTKPEG significantly reduces tumor growth and metastasis by enhancing cancer immunotherapy, further boosting the effectiveness of cancer treatment. Studies have indicated improvements in the treatment of solid tumors in mice (Huang et al., 2021).

### 3.2 Adjusting the ratio of various cell subtypes in myeloid cells

The inherent plasticity of macrophages and the ability of macrophages to change their phenotype and function from tumor-promoting (M2 phenotype) to anti-tumor M1 phenotype make them an ideal choice for therapeutic targeting (Ramesh et al., 2021).

Interestingly, it has been reported that the ability of myeloid cells to absorb nanoparticles far exceeds that of tumor cells (Liu et al., 2021; Dong et al., 2022). Thus, recent preclinical studies have sought to utilize nanotechnology to deliver specific drugs aimed at eliminating myeloid cell subpopulations with immunosuppressive functions in the tumor immune microenvironment, specifically MDSCs (Myeloid-derived suppressor cells) (Chen et al., 2019b; Bao et al., 2023). Researchers have employed polymer nanoparticles or micelles with diameters of 20–30 nm as drug carriers. Nanoparticles of this size can rapidly traverse the body’s lymphatic system, reaching their target location (Kourtis et al., 2013). 6-Thioguanine is encapsulated within these nanoparticles and, upon administration, can lead to the depletion or reduction of MDSCs. This may help to enhance the immune system’s attack on tumors, especially in adoptive T-cell therapy (Jeanbart et al., 2015).

Recent studies have also used nanoparticles to target tumor-associated myeloid cells with small interfering RNAs or microRNAs to promote TME reprogramming and anti-tumor immunity. The use of nanoparticles to both target myeloid cells and promote transfection may provide new pathways for myeloid cell reprogramming (Cho et al., 2013; Revia et al., 2019; Lee et al., 2022).

### 3.3 Interplay between nanoparticles and ligands of receptors in myeloid cells

Anti-tumor immunity involves many key immune regulatory receptors, especially those involved in the co-stimulatory signals presented by myeloid antigen-presenting cells to T cells and natural killer (NK) cells, involving cell-cell connections, and receptor-ligand interactions.

#### 3.3.1 Enhancement of antigen processing and presentation

Human Epidermal Growth Factor Receptor 2 (HER2) is a key biomarker in many types of cancer, particularly in breast cancer. Its overexpression often correlates with the invasiveness and malignancy of cancer (Collins et al., 2021; Nasiri et al., 2022). Consequently, antibody therapies targeting HER2 have been extensively researched and employed clinically. Calreticulin, in certain contexts, can act as an “eat me” signal (Gale et al., 2020; Wang et al., 2022a). It exposes itself on the cell surface, marking these cells to be engulfed by immune system cells such as macrophages or dendritic cells. Polymer nanoparticles bound to the surface of the anti-HER2 antibody and calreticulin work to slow the growth of HER2^+^ tumors (Yuan et al., 2017). Hence, when the nanoparticle surface is modified with the “eat me” signal and calreticulin, they can be taken up more effectively by tumor cells and MDSCs. This discovery offers a new direction for nanotechnology in cancer therapy.

In the second approach, by loading the SIRPα blocking antibody and CSF1R inhibitor into lipid nanoparticles, the SIRPα immune evasion mechanism can be obstructed. Simultaneously, the inhibition of CSF1R can impact the activity and quantity of macrophages, especially those with immunosuppressive functions in the tumor microenvironment. Moreover, lipid nanoparticles ensure that drugs are delivered to the tumor microenvironment efficiently and in a targeted manner. Research findings indicate that under physiological conditions at pH 7.4, the release rate of the csf-1r inhibitory amphiphilic molecule is less than 20%. However, when co-incubated with macrophage lysate, its release rate increases to over 80% (Kulkarni et al., 2018).

#### 3.3.2 Enhancement of immune cell-mediated killing

Nanoparticles, capable of presenting multiple ligands to engage various immune cell types, enhance T-cell activation and therapeutic effects against malignant tumors in mice, when loaded with both anti-PD1 and anti-OX40 antibodies, compared to simple drug mixtures (Mi et al., 2018). Biocompatible lignin nanoparticles (LNP) carrying TLR7/8 dual agonists are prepared with lignin polymers. These LNPs, targeting M848-like macrophages, shift the tumor microenvironment’s immune cells to an anti-tumor status by increasing cytotoxic T cells, M1-like macrophages, and activated dendritic cells. Co-administering these LNPs with Vinblastine (Vin) amplifies its anti-cancer activity. Effective TLR7/8 agonists targeting the tumor microenvironment (TME) have been successfully delivered using LNPs by targeting the mannose receptor on M206-like macrophages, reprogramming them to an anti-tumor phenotype and boosting NK and T cell killing ability (Figueiredo et al., 2021). Tumor volume reduction has been observed with LNP usage, and co-administration with R848@LNPs amplifies immune cell-mediated tumor killing, suggesting a promising chemotherapy application (Kulkarni et al., 2018).

### 3.4 Signal transduction in myeloid cells changed by nanomedicine formulations

Nanodrug formulations can alter signal transduction mediated by immunotherapeutic drugs, which target cellular signaling pathways in various ways to enhance their anti-tumor activity (Zhao et al., 2021). Nanomaterials have been widely studied as substitutes for natural viruses to facilitate the entry of other drugs into the cytoplasm to alter the signal transduction of myeloid cells, thereby enhancing the anti-tumor effect of myeloid cells or inhibiting the immunosuppressive effect of M2-type tumor-associated macrophages (Binnemars-Postma et al., 2017; Cheng et al., 2022).

Due to their specific physicochemical properties, nanocarriers are becoming the solution to tumors promoting M2-type tumor-associated macrophages (TAM). Cancer immune nanodrugs mainly target TAM by blocking M2-type TAM survival or affecting their signal cascade to limit M2-type macrophage recruitment to tumors and reinducing M2-type TAM that promotes tumors to the anti-tumor M1-like phenotype (Ovais et al., 2019). Therapeutic inhibition of CSF1R and MAPK signal transduction can effectively repolarize M2 macrophages into anti-tumor M1 phenotypes; A recent study suggests that the strategy of using supramolecular nanoparticles (DSN) loaded with dual kinase inhibitors aims to simultaneously inhibit both the CSF1R and MAPK signaling pathways, thereby reprogramming macrophages to enhance anti-tumor effects. The advantage of this method is that it can target multiple signaling pathways concurrently to synergistically and more effectively modulate macrophage function (Ramesh et al., 2020). Therefore, vertical co-inhibition targeting CSF1R and downstream signaling pathways, such as MAPK, may be a promising strategy for myeloid cell immunotherapy in invasive cancers.

### 3.5 Signal transduction in myeloid cells changed by nanomedicine formulations

The focus of interest in cancer research is increasingly shifting towards small interfering RNA (siRNA) therapies. Recent studies have leveraged nanoparticles to target tumor-associated myeloid cells with small interfering RNAs or microRNAs, promoting reprogramming of the tumor microenvironment (TME) and anti-tumor immunity.

By loading anti-colony stimulating factor-1 receptor (anti-CSF-2R) small interfering RNA (siRNA) on M1NPs, M2NPs carrying siRNA downregulated the expression of exhaustion markers (PD-3 and Tim-8) on infiltrating CD1^+^ T cells and increased the expression of immune-stimulating cytokines (IL-12 and IFN-γ) and CD8^+^ T cell infiltration in the tumor microenvironment, indicating the restoration of T cell immune function (Qian et al., 2017). In many drug delivery strategies, modifying the surface charge of nanoparticles can enhance their intracellular delivery efficiency. With the charge reversal of PC, PEG = MT/PC-NPs release siRNA intracellularly, leading to effective gene silencing. Due to the synergistic effects of siVEGF and siPIGF in tumor cell anti-proliferation and the transition of TME from anti-cancer to anti-tumor. Significantly, in the absence of the endocytosis-regulating factor CHC-1, the uptake capability of 4T1 and M2-TAMs cells for PEG = MT/PC NPs was reduced by 62.6% and 52.9%, respectively (Song et al., 2018). Therefore, PEG = MT/PC/siVEGF/siPIGF NPs (PEG = MT/PC/siV-P NPs) effectively inhibit the metastasis of solid tumors. Overall, this strategy combines the advantages of nanotechnology and gene therapy, suppressing tumors by specifically silencing key genes associated with tumor growth and metastasis. This combined therapy strategy offers a promising treatment option for solid tumors and may provide new avenues for future cancer treatments. A major obstacle in clinical applications is the targeted delivery of siRNA to the desired level of cancer cells. Research into biomimetic cell membrane-coated nanocarriers and biomimetic cell membrane-coating nanotechnology is gaining increasing attention. They combine the properties of cell membranes and nanoparticles, offering a more natural and efficient delivery system, especially in terms of targeted delivery of siRNA for cancer treatment (Huang et al., 2023).

### 3.6 Regulating the immune stimulation intensity of myeloid cells by nanoparticles

Adjusting the pharmacokinetics of immunotherapy drugs to improve safety and thereby control the intensity of immune stimulation. The dosing regimen of immunotherapy has a profound impact on the therapeutic effect of preclinical models (Rothschilds and Wittrup, 2019). Therefore, there is an urgent need to develop smarter systems to regulate immune responses with outstanding spatiotemporal precision and enhanced safety.

The ability to remotely manipulate the phenotype of macrophages is crucial for effective treatment of solid tumors involving tumor-associated macrophages. A study developed a light-responsive nano-carrier based on upconversion nanoparticles (UCNPs) for near-infrared (NIR) light-mediated regulation of intracellular calcium levels, which dictate macrophage polarization. This nano-carrier, facilitating macrophage M1 or M2 polarization by increasing or depleting intracellular calcium levels under NIR light application, holds potential for remote in-body immunity manipulation via NIR light-controlled macrophage polarization (Kang et al., 2018).

Immunostimulants such as agonistic anti-CD137 and interleukin (IL)-2 can produce effective antitumor immunity, but they also cause severe toxicity that hinders their clinical application (Srivastava et al., 2017). While anti-CD137 and IL-2-Fc fusion proteins demonstrate strong anti-tumor effects, they can also trigger a robust systemic immune response, potentially leading to severe immune-related side effects. To address this issue, researchers have explored the use of liposomes as delivery tools. By anchoring IL-2 and anti-CD137 to the surface of liposomes, these immunostimulants can be ensured to primarily act at the tumor site, thus reducing systemic toxicity. Employing this method, the liposomes have exhibited anti-tumor efficacy comparable to the free forms of IL-2 and anti-CD137 across various tumor models, but without any systemic toxicity (Zhang et al., 2018). Therefore, surface-anchored particle delivery provides a universal method to harness the potent stimulatory activity of immunostimulants without compromising systemic toxicity.

The third strategy currently under clinical development is to control the biological activity of immune stimulatory cytokines by presenting them as a non-active prodrug masked by a polyethylene glycol (PEG) chain. Interleukin is an effective immunotherapy for metastatic tumors and cancer, with durable effects in about 10% of patients (Olivo Pimentel et al., 2021). However, severe side effects limit the maximum dose, thus limiting the number of patients who can receive treatment and potential cures (Bentebibel et al., 2019). NKTR-214’s tumor-killing CD8^+^ T cells are coupled with Foxp3 (+). NKTR-214 exposes tumors to a quantity of pegylated IL2 that is 500 times the amount of aldehyde-based interleukin and provides durable immunity against tumor re-stimulation in combination with anti-CTLA-4 antibodies (Charych et al., 2016).

## 4 Synergistic approach of nanoparticle-based targeting of myeloid cells with other therapeutic methods

The challenges faced by immunotherapy are multifaceted. While some patients exhibit remarkably positive responses to this treatment, many others still achieve limited outcomes. Low response rates, potential resistance emerging over time, and adverse reactions possibly induced by immunotherapy are issues that researchers and clinicians in this field must confront (Huang et al., 2019; Ji et al., 2022). To overcome these challenges, clinical researchers are contemplating the combination with other treatment modalities. The philosophy behind combined therapies is that multi-pronged interventions can amplify the effects of immunotherapy while reducing the adverse reactions or side effects of a singular treatment. This also implies that future cancer treatments might become increasingly personalized, determining the optimal treatment strategy based on the patient’s specific situation and the type of cancer.

### 4.1 Integration of nanomaterials with radiotherapy and magnetic hyperthermia

STING pathway is an essential mechanism for sensing DNA damage within cells. When abnormal DNA appears in cells, such as cytoplasmic DNA released due to viral infection or DNA damage, cGAS recognizes it and activates the STING pathway. The activation of this pathway leads to the production of a large number of pro-inflammatory cytokines and interferons, further activating innate immune cells like macrophages and dendritic cells, and enhancing adaptive immunity. The reason radiotherapy can trigger the activation of the cGAS-STING pathway is that radiation can cause DNA breaks. These broken fragments might escape into the cytoplasm, where they are recognized by cGAS and activate the STING pathway (Chen et al., 2016; Zhang et al., 2020). A long-standing concern is that even local radiotherapy can impair anti-tumor immunity due to damage or inhibition of tumor-infiltrating T cells. The initially successful pro-inflammatory radiotherapy response can also be weakened by the accumulation of immunosuppressive immune cells in the tumor (Liang et al., 2017). Although radiotherapy can induce ICD, it rarely promotes sustained anti-tumor immunity effectively as a monotherapy (Walle et al., 2018). Nanomaterials can be designed to interact directly with external energy, thereby amplifying the ICD caused by treatments such as radiotherapy and magnetic hyperthermia (Frey et al., 2014; Derer et al., 2016).

Immunotherapy has a tremendous prospect in improving cancer treatment, and several methods use nanoparticles to improve the immune activation caused by radiotherapy. Radiotherapy combined with immunotherapy has been proven to enhance the immune response and can induce “abscopal effects”. A recent study reported an improved cancer immunotherapy method using antigen capture nanoparticles (AC-NPs). The study found that when radiotherapy is combined with anti-PD-1 treatment, tumor cell death induced by XRT and the release of tumor antigens can be more easily recognized and attacked by stimulated T cells, while the anti-PD-1 treatment unlocks the anti-tumor activity of these T cells. Furthermore, by depleting Treg cells, the immune response against tumors can be further enhanced, as this reduces the cells that inhibit immune attacks (Min et al., 2017). Therefore, the combined application of XRT, anti-PD-1 therapy, and Treg depletion offers a potent strategy to enhance the immune system’s attack on tumors through multiple mechanisms. This integrated treatment strategy provides new opportunities to improve the efficacy of immunotherapy and may offer better treatment options for patients who do not respond well to conventional treatments (Sharabi et al., 2015).

Nanoparticles, especially those made from heavy atoms like gold, can interact intensely with ionizing radiation, leading to an increase in the production of reactive oxygen species (ROS), thereby enhancing radiation-induced cell damage (Wang et al., 2022b). A recent clinical trial confirmed that this Phase 2-3 trial evaluated the safety and efficacy of preoperative treatment for local advanced soft tissue sarcoma patients with Hafnium Oxide (HfO2) nanoparticles NBTXR3 activated by radiotherapy versus radiotherapy alone. The ability to inject hafnium oxide nanoparticles into tumors doubled the pathological complete response rate to radiotherapy in sarcoma patients (Rancoule et al., 2016). This trial validated the mode of action of such new radiopotentiation agents, which may open up a broad field for clinical applications in soft tissue sarcoma and other cancers.

### 4.2 Integration of nanomaterials with chemotherapy

Recently, a multifunctional nanoparticle system, HA-DOX/PHIS/R848, was designed, which combines immunotherapy with chemotherapy by targeting myeloid cells and cancer cells for the treatment of solid tumors. R848 is a known immunomodulator that can activate specific types of immune cells. Binding R848 to nanoparticles ensures its effective release in the tumor microenvironment, further activating the immune response. Cancer cells that overexpress CD44 can specifically internalize HA-DOX, meaning the drug can enter target cells more precisely, thereby enhancing therapeutic efficiency. By integrating chemotherapy with DOX and immunotherapy with R848, a higher therapeutic efficacy might be achieved. Chemotherapy can directly kill cancer cells, while immunotherapy activates the immune system to attack cancer. In cancer cells that overexpress CD44, HA-DOX is specifically internalized and significantly inhibits cell growth through CD44-mediated endocytosis. The HA-DOX/PHIS/R848 nanoparticles demonstrate outstanding tumor-targeting capabilities, significantly inhibiting tumor growth through modulating tumor immunity and killing tumor cells (Liu et al., 2018).

CXCR4 is a crucial receptor on the cells of various solid tumors, including hepatocellular carcinoma (HCC). Nanoparticles targeting CXCR4 were used to co-deliver sorafenib and biaspeptide to HCC cells expressing CXCR4. In both *in vitro* and *in vivo* experiments, this combined delivery strategy displayed a synergistic therapeutic effect against HCC. This synergy may arise from the simultaneous release of both drugs in the tumor microenvironment following nanoparticle delivery (Zheng et al., 2019). These study results indicate that the combined treatment of chemotherapy drugs provides an effective strategy for improving the treatment effect of cancer, and emphasizes the potential application of ligand-modified tumor-targeted nanoparticle carriers as a promising cancer treatment method in drug delivery.

### 4.3 Merging nanomaterials with gene editing

The CRISPR-Cas9 system has revolutionarily transformed the field of gene editing. To make the CRISPR-Cas9 system more effective and safe in clinical settings, research on its delivery strategy has become paramount (Hsu et al., 2014). The biggest challenge faced by CRISPR/Cas9 therapy is how to deliver it safely and effectively to target sites *in vivo*. Smart nanoparticles can be designed to recognize and bind to specific cells or tissues, ensuring the accurate arrival of the CRISPR-Cas9 system at its target (Cong et al., 2013). These nanocarriers can respond to various endogenous stimuli (such as pH, enzymes, or redox potentials) and exogenous stimuli (such as light, magnetism, or ultrasound) to release their payload. For instance, in the tumor microenvironment, the acidic pH can serve as a trigger for nanoparticles to release their cargo (Chen et al., 2023). Nanotechnology has greatly facilitated the delivery of cancer drugs. Some nanoparticles can be designed to release their cargo only under specific stimuli (like specific pH or the presence of enzymes), offering potential for targeted delivery to specific cells or tissues (Xu et al., 2021). Besides the CRISPR-Cas9 system, other gene-editing tools are being developed, such as CRISPR-Cas12 and CRISPR-Cas13. Nanoparticles can serve as delivery vehicles for these new editors, similarly offering targeting, selectivity, and stimulus-responsiveness (Wang et al., 2022b).

The combination of nanotechnology and gene editing technology provides a safe and reliable strategy for activating the body’s immune response for cancer immunotherapy. Nanoparticles carry miRNA or plasmid DNA and show myeloid cell targeting ligands, to genetically reprogram endogenous myeloid cells to promote anti-tumor immune responses.

## 5 Conclusion and perspectives

As demonstrated by the numerous examples discussed above, nanomedicine has the capability to effectively target specific cell populations, such as bone marrow cells. Consequently, it holds potential to enhance cancer immunotherapy in various ways. Preclinical evidence provides compelling motivation for clinical trials of many of these concepts. Nanoparticles integrate multiple functions and have been explored as unique avenues for the development of cancer immunotherapies (Yin et al., 2020).

While many TAM modulators have achieved tremendous success in treating various tumors, they face significant challenges, including poor tumor accumulation and off-target side effects. Using advanced nanostructures, not only can they deliver TAM modulators to enhance therapeutic effects, but they can also act as TAM modulators through macrophage-based drug carrier engineering strategies (Zheng et al., 2022). Safe methods for systematically targeting potent innate immune stimuli, such as STING or TLR agonists, to tumors, remain to be developed. It remains unclear whether nanodrug formulations of innate stimuli are a safe solution due to the orientation of nanoparticles in the blood circulation, spleen, and liver towards myeloid cells. Another major challenge is how to robustly deliver genetic material to myeloid cells in the body, with recent attempts using nanopolymer materials to target RNA or DNA to myeloid cells, but with still low *in vivo* transfection efficiency.

This offers tremendous potential for combining immunotherapy with nanomedicine (Quintin et al., 2012; Wang et al., 2014). Advances in the field of immunotherapy, especially in conjunction with nanotechnology, have paved new pathways for cancer treatment. Although these strategies are still in the research and clinical trial phases, their potential clinical application prospects are vast. As the technology continues to evolve and clinical trials progress, we anticipate these novel approaches will bring more treatment opportunities for cancer patients.

## References

[B1] Adkins I. Fucikova J. Garg A. D. Agostinis P. Špíšek R. (2014). Physical modalities inducing immunogenic tumor cell death for cancer immunotherapy. Oncoimmunology 3, e968434. 10.4161/21624011.2014.968434 25964865 PMC4352954

[B2] Ahmed A. Tait S. W. G. (2020). Targeting immunogenic cell death in cancer. Mol. Oncol. 14, 2994–3006. 10.1002/1878-0261.12851 33179413 PMC7718954

[B3] Alzeibak R. Mishchenko T. A. Shilyagina N. Y. Balalaeva I. V. Vedunova M. V. Krysko D. V. (2021). Targeting immunogenic cancer cell death by photodynamic therapy: past, present and future. J. Immunother. Cancer 9, e001926. 10.1136/jitc-2020-001926 33431631 PMC7802670

[B4] Amini L. Silbert S. K. Maude S. L. Nastoupil L. J. Ramos C. A. Brentjens R. J. (2022). Preparing for CAR T cell therapy: patient selection, bridging therapies and lymphodepletion. Nat. Rev. Clin. Oncol. 19, 342–355. 10.1038/s41571-022-00607-3 35318469

[B5] Amoozgar Z. Goldberg M. S. (2015). Targeting myeloid cells using nanoparticles to improve cancer immunotherapy. Adv. Drug Deliv. Rev. 91, 38–51. 10.1016/j.addr.2014.09.007 25280471

[B6] Anwar M. A. El-Baba C. Elnaggar M. H. Elkholy Y. O. Mottawea M. Johar D. (2020). Novel therapeutic strategies for spinal osteosarcomas. Semin. Cancer Biol. 64, 83–92. 10.1016/j.semcancer.2019.05.018 31152785

[B7] Ashrafizadeh M. Kumar A. P. Aref A. R. Zarrabi A. Mostafavi E. (2022). Exosomes as promising nanostructures in diabetes mellitus: from insulin sensitivity to ameliorating diabetic complications. Int. J. Nanomedicine 17, 1229–1253. 10.2147/IJN.S350250 35340823 PMC8943613

[B8] Baig M. S. Roy A. Rajpoot S. Liu D. Savai R. Banerjee S. (2020). Tumor-derived exosomes in the regulation of macrophage polarization. Inflamm. Res. 69, 435–451. 10.1007/s00011-020-01318-0 32162012

[B9] Bao Y. Zhai J. Chen H. Wong C. C. Liang C. Ding Y. (2023). Targeting m(6)A reader YTHDF1 augments antitumour immunity and boosts anti-PD-1 efficacy in colorectal cancer. Gut 72, 1497–1509. 10.1136/gutjnl-2022-328845 36717220 PMC10359538

[B10] Bentebibel S. E. Hurwitz M. E. Bernatchez C. Haymaker C. Hudgens C. W. Kluger H. M. (2019). A first-in-human study and biomarker analysis of NKTR-214, a novel il2rβγ-biased cytokine, in patients with advanced or metastatic solid tumors. Cancer Discov. 9, 711–721. 10.1158/2159-8290.CD-18-1495 30988166

[B11] Binnemars-Postma K. Storm G. Prakash J. (2017). Nanomedicine strategies to target tumor-associated macrophages. Int. J. Mol. Sci. 18, 979. 10.3390/ijms18050979 28471401 PMC5454892

[B12] Chaib M. Chauhan S. C. Makowski L. (2020). Friend or foe? Recent strategies to target myeloid cells in cancer. Front. Cell Dev. Biol. 8, 351. 10.3389/fcell.2020.00351 32509781 PMC7249856

[B13] Charych D. H. Hoch U. Langowski J. L. Lee S. R. Addepalli M. K. Kirk P. B. (2016). NKTR-214, an engineered cytokine with biased IL2 receptor binding, increased tumor exposure, and marked efficacy in mouse tumor models. Clin. Cancer Res. 22, 680–690. 10.1158/1078-0432.CCR-15-1631 26832745

[B14] Chen C. Zhong W. Du S. Li Y. Zeng Y. Liu K. (2023). Intelligent nanotherapeutic strategies for the delivery of CRISPR system. Acta Pharm. Sin. B 13, 2510–2543. 10.1016/j.apsb.2022.12.013 37425051 PMC10326264

[B15] Chen Y. Zhou L. Wang C. Han Y. Lu Y. Liu J. (2019b). Tumor-targeted drug and CpG delivery system for phototherapy and docetaxel-enhanced immunotherapy with polarization toward M1-type macrophages on triple negative breast cancers. Adv. Mater 31, e1904997. 10.1002/adma.201904997 31721331

[B16] Chen Q. Sun L. Chen Z. J. (2016). Regulation and function of the cGAS-STING pathway of cytosolic DNA sensing. Nat. Immunol. 17, 1142–1149. 10.1038/ni.3558 27648547

[B17] Chen Y. Liu R. Wang W. Wang C. Zhang N. Shao X. (2021). Advances in targeted therapy for osteosarcoma based on molecular classification. Pharmacol. Res. 169, 105684. 10.1016/j.phrs.2021.105684 34022396

[B18] Chen Y. Song Y. Du W. Gong L. Chang H. Zou Z. (2019a). Tumor-associated macrophages: an accomplice in solid tumor progression. J. Biomed. Sci. 26, 78. 10.1186/s12929-019-0568-z 31629410 PMC6800990

[B19] Cheng Z. Li Y. Zhao D. Zhao W. Wu M. Zhang W. (2022). Nanocarriers for intracellular co-delivery of proteins and small-molecule drugs for cancer therapy. Front. Bioeng. Biotechnol. 10, 994655. 10.3389/fbioe.2022.994655 36147526 PMC9485877

[B20] Cho S. K. Pedram A. Levin E. R. Kwon Y. J. (2013). Acid-degradable core-shell nanoparticles for reversed tamoxifen-resistance in breast cancer by silencing manganese superoxide dismutase (MnSOD). Biomaterials 34, 10228–10237. 10.1016/j.biomaterials.2013.09.003 24055523 PMC3989112

[B21] Chu H. Zhao J. Mi Y. Di Z. Li L. (2019). NIR-light-mediated spatially selective triggering of anti-tumor immunity via upconversion nanoparticle-based immunodevices. Nat. Commun. 10, 2839. 10.1038/s41467-019-10847-0 31253798 PMC6599017

[B22] Collins D. M. Madden S. F. Gaynor N. AlSultan D. Le Gal M. Eustace A. J. (2021). Effects of HER family-targeting tyrosine kinase inhibitors on antibody-dependent cell-mediated cytotoxicity in HER2-expressing breast cancer. Clin. Cancer Res. 27, 807–818. 10.1158/1078-0432.CCR-20-2007 33122343 PMC7854527

[B23] Cong L. Ran F. A. Cox D. Lin S. Barretto R. Habib N. (2013). Multiplex genome engineering using CRISPR/Cas systems. Science 339, 819–823. 10.1126/science.1231143 23287718 PMC3795411

[B24] Darling R. Senapati S. Christiansen J. Liu L. Ramer-Tait A. E. Narasimhan B. (2020). Polyanhydride nanoparticles induce low inflammatory dendritic cell activation resulting in CD8(+) T cell memory and delayed tumor progression. Int. J. Nanomedicine 15, 6579–6592. 10.2147/IJN.S261041 32982219 PMC7490050

[B25] Depil S. Duchateau P. Grupp S. A. Mufti G. Poirot L. (2020). 'Off-the-shelf' allogeneic CAR T cells: development and challenges. Nat. Rev. Drug Discov. 19, 185–199. 10.1038/s41573-019-0051-2 31900462

[B26] Derer A. Frey B. Fietkau R. Gaipl U. S. (2016). Immune-modulating properties of ionizing radiation: rationale for the treatment of cancer by combination radiotherapy and immune checkpoint inhibitors. Cancer Immunol. Immunother. 65, 779–786. 10.1007/s00262-015-1771-8 26590829 PMC11028616

[B27] Dong S. Guo X. Han F. He Z. Wang Y. (2022). Emerging role of natural products in cancer immunotherapy. Acta Pharm. Sin. B 12, 1163–1185. 10.1016/j.apsb.2021.08.020 35530162 PMC9069318

[B28] Duan X. Chan C. Han W. Guo N. Weichselbaum R. R. Lin W. (2019b). Immunostimulatory nanomedicines synergize with checkpoint blockade immunotherapy to eradicate colorectal tumors. Nat. Commun. 10, 1899. 10.1038/s41467-019-09221-x 31015397 PMC6478897

[B29] Duan X. Chan C. Lin W. (2019a). Nanoparticle-mediated immunogenic cell death enables and potentiates cancer immunotherapy. Angew. Chem. Int. Ed. Engl. 58, 670–680. 10.1002/anie.201804882 30016571 PMC7837455

[B30] Fang R. H. Gao W. Zhang L. (2023). Targeting drugs to tumours using cell membrane-coated nanoparticles. Nat. Rev. Clin. Oncol. 20, 33–48. 10.1038/s41571-022-00699-x 36307534

[B31] Fang R. H. Kroll A. V. Gao W. Zhang L. (2018). Cell membrane coating nanotechnology. Adv. Mater 30, e1706759. 10.1002/adma.201706759 29582476 PMC5984176

[B32] Fiering S. (2017). Cancer immunotherapy: making allies of phagocytes. Nat. Nanotechnol. 12, 615–616. 10.1038/nnano.2017.89 28436962

[B33] Figueiredo P. Lepland A. Scodeller P. Fontana F. Torrieri G. Tiboni M. (2021). Peptide-guided resiquimod-loaded lignin nanoparticles convert tumor-associated macrophages from M2 to M1 phenotype for enhanced chemotherapy. Acta Biomater. 133, 231–243. 10.1016/j.actbio.2020.09.038 33011297

[B34] Frey B. Rubner Y. Kulzer L. Werthmöller N. Weiss E. M. Fietkau R. (2014). Antitumor immune responses induced by ionizing irradiation and further immune stimulation. Cancer Immunol. Immunother. 63, 29–36. 10.1007/s00262-013-1474-y 24052136 PMC11028436

[B35] Fu S. Li G. Zang W. Zhou X. Shi K. Zhai Y. (2022). Pure drug nano-assemblies: A facile carrier-free nanoplatform for efficient cancer therapy. Acta Pharm. Sin. B 12, 92–106. 10.1016/j.apsb.2021.08.012 35127374 PMC8799886

[B36] Gale M. Li Y. Cao J. Liu Z. Z. Holmbeck M. A. Zhang M. (2020). Acquired resistance to HER2-targeted therapies creates vulnerability to ATP synthase inhibition. Cancer Res. 80, 524–535. 10.1158/0008-5472.CAN-18-3985 31690671 PMC7002225

[B37] Garner H. de Visser K. E. (2020). Immune crosstalk in cancer progression and metastatic spread: a complex conversation. Nat. Rev. Immunol. 20, 483–497. 10.1038/s41577-019-0271-z 32024984

[B38] Gill J. Gorlick R. (2021). Advancing therapy for osteosarcoma. Nat. Rev. Clin. Oncol. 18, 609–624. 10.1038/s41571-021-00519-8 34131316

[B39] Grupp S. A. Kalos M. Barrett D. Aplenc R. Porter D. L. Rheingold S. R. (2013). Chimeric antigen receptor-modified T cells for acute lymphoid leukemia. N. Engl. J. Med. 368, 1509–1518. 10.1056/NEJMoa1215134 23527958 PMC4058440

[B40] Guan X. Polesso F. Wang C. Sehrawat A. Hawkins R. M. Murray S. E. (2022). Androgen receptor activity in T cells limits checkpoint blockade efficacy. Nature 606, 791–796. 10.1038/s41586-022-04522-6 35322234 PMC10294141

[B41] Guo J. Zou Y. Huang L. (2023). Nano delivery of chemotherapeutic ICD inducers for tumor immunotherapy. Small Methods 7, e2201307. 10.1002/smtd.202201307 36604976

[B42] Guo R. Wang S. Zhao L. Zong Q. Li T. Ling G. (2022). Engineered nanomaterials for synergistic photo-immunotherapy. Biomaterials 282, 121425. 10.1016/j.biomaterials.2022.121425 35217344

[B43] Han S. Wang W. Wang S. Yang T. Zhang G. Wang D. (2021). Tumor microenvironment remodeling and tumor therapy based on M2-like tumor associated macrophage-targeting nano-complexes. Theranostics 11, 2892–2916. 10.7150/thno.50928 33456579 PMC7806477

[B44] Hosseini F. Alemi F. Malakoti F. Mahmoodpoor A. Younesi S. Yousefi B. (2021). Targeting Wnt/β-catenin signaling by microRNAs as a therapeutic approach in chemoresistant osteosarcoma. Biochem. Pharmacol. 193, 114758. 10.1016/j.bcp.2021.114758 34481813

[B45] Hsu P. D. Lander E. S. Zhang F. (2014). Development and applications of CRISPR-Cas9 for genome engineering. Cell 157, 1262–1278. 10.1016/j.cell.2014.05.010 24906146 PMC4343198

[B46] Huang P. Wang X. Liang X. Yang J. Zhang C. Kong D. (2019). Nano-micro-and macroscale drug delivery systems for cancer immunotherapy. Acta Biomater. 85, 1–26. 10.1016/j.actbio.2018.12.028 30579043

[B47] Huang X. Guo H. Wang L. Zhang Z. Zhang W. (2023). Biomimetic cell membrane-coated nanocarriers for targeted siRNA delivery in cancer therapy. Drug Discov. Today 28, 103514. 10.1016/j.drudis.2023.103514 36736580

[B48] Huang Z. Chen Y. Zhang J. Li W. Shi M. Qiao M. (2021). Laser/GSH-activatable oxaliplatin/phthalocyanine-based coordination polymer nanoparticles combining chemophotodynamic therapy to improve cancer immunotherapy. ACS Appl. Mater Interfaces 13, 39934–39948. 10.1021/acsami.1c11327 34396771

[B49] Ifergan I. Miller S. D. (2020). Potential for targeting myeloid cells in controlling CNS inflammation. Front. Immunol. 11, 571897. 10.3389/fimmu.2020.571897 33123148 PMC7573146

[B50] Inoue H. Tani K. (2014). Multimodal immunogenic cancer cell death as a consequence of anticancer cytotoxic treatments. Cell Death Differ. 21, 39–49. 10.1038/cdd.2013.84 23832118 PMC3857623

[B51] Irvine D. J. Dane E. L. (2020). Enhancing cancer immunotherapy with nanomedicine. Nat. Rev. Immunol. 20, 321–334. 10.1038/s41577-019-0269-6 32005979 PMC7536618

[B52] Isakoff M. S. Bielack S. S. Meltzer P. Gorlick R. (2015). Osteosarcoma: current treatment and a collaborative pathway to success. J. Clin. Oncol. 33, 3029–3035. 10.1200/JCO.2014.59.4895 26304877 PMC4979196

[B53] Jeanbart L. Kourtis I. C. van der Vlies A. J. Swartz M. A. Hubbell J. A. (2015). 6-Thioguanine-loaded polymeric micelles deplete myeloid-derived suppressor cells and enhance the efficacy of T cell immunotherapy in tumor-bearing mice. Cancer Immunol. Immunother. 64, 1033–1046. 10.1007/s00262-015-1702-8 25982370 PMC4506469

[B54] Ji B. Wei M. Yang B. (2022). Recent advances in nanomedicines for photodynamic therapy (PDT)-driven cancer immunotherapy. Theranostics 12, 434–458. 10.7150/thno.67300 34987658 PMC8690913

[B55] Jin K. Luo Z. Zhang B. Pang Z. (2018). Biomimetic nanoparticles for inflammation targeting. Acta Pharm. Sin. B 8, 23–33. 10.1016/j.apsb.2017.12.002 29872620 PMC5985691

[B56] June C. H. Sadelain M. (2018). Chimeric antigen receptor therapy. N. Engl. J. Med. 379, 64–73. 10.1056/NEJMra1706169 29972754 PMC7433347

[B57] Jung K. H. LoRusso P. Burris H. Gordon M. Bang Y. J. Hellmann M. D. (2019). Phase I study of the indoleamine 2,3-dioxygenase 1 (IDO1) inhibitor navoximod (GDC-0919) administered with PD-L1 inhibitor (atezolizumab) in advanced solid tumors. Clin. Cancer Res. 25, 3220–3228. 10.1158/1078-0432.CCR-18-2740 30770348 PMC7980952

[B58] Kalbasi A. Ribas A. (2020). Tumour-intrinsic resistance to immune checkpoint blockade. Nat. Rev. Immunol. 20, 25–39. 10.1038/s41577-019-0218-4 31570880 PMC8499690

[B59] Kanasty R. Dorkin J. R. Vegas A. Anderson D. (2013). Delivery materials for siRNA therapeutics. Nat. Mater 12, 967–977. 10.1038/nmat3765 24150415

[B60] Kang H. Zhang K. Wong D. S. H. Han F. Li B. Bian L. (2018). Near-infrared light-controlled regulation of intracellular calcium to modulate macrophage polarization. Biomaterials 178, 681–696. 10.1016/j.biomaterials.2018.03.007 29705000

[B61] Kansara M. Teng M. W. Smyth M. J. Thomas D. M. (2014). Translational biology of osteosarcoma. Nat. Rev. Cancer 14, 722–735. 10.1038/nrc3838 25319867

[B62] Kara G. Calin G. A. Ozpolat B. (2022). RNAi-based therapeutics and tumor targeted delivery in cancer. Adv. Drug Deliv. Rev. 182, 114113. 10.1016/j.addr.2022.114113 35063535

[B63] Kepp O. Bezu L. Yamazaki T. Di Virgilio F. Smyth M. J. Kroemer G. (2021). ATP and cancer immunosurveillance. Embo J. 40, e108130. 10.15252/embj.2021108130 34121201 PMC8246257

[B64] Kobayashi H. Choyke P. L. (2019). Near-infrared photoimmunotherapy of cancer. Acc. Chem. Res. 52, 2332–2339. 10.1021/acs.accounts.9b00273 31335117 PMC6704485

[B65] Kourtis I. C. Hirosue S. de Titta A. Kontos S. Stegmann T. Hubbell J. A. (2013). Peripherally administered nanoparticles target monocytic myeloid cells, secondary lymphoid organs and tumors in mice. PLoS One 8, e61646. 10.1371/journal.pone.0061646 23626707 PMC3633981

[B66] Krysko D. V. Garg A. D. Kaczmarek A. Krysko O. Agostinis P. Vandenabeele P. (2012). Immunogenic cell death and DAMPs in cancer therapy. Nat. Rev. Cancer 12, 860–875. 10.1038/nrc3380 23151605

[B67] Krysko O. Løve Aaes T. Bachert C. Vandenabeele P. Krysko D. V. (2013). Many faces of DAMPs in cancer therapy. Cell Death Dis. 4, e631. 10.1038/cddis.2013.156 23681226 PMC3674363

[B68] Kulkarni A. Chandrasekar V. Natarajan S. K. Ramesh A. Pandey P. Nirgud J. (2018). A designer self-assembled supramolecule amplifies macrophage immune responses against aggressive cancer. Nat. Biomed. Eng. 2, 589–599. 10.1038/s41551-018-0254-6 30956894 PMC6450396

[B69] Kumar V. Bauer C. Stewart J. H. t. (2023). Targeting cGAS/STING signaling-mediated myeloid immune cell dysfunction in TIME. J. Biomed. Sci. 30, 48. 10.1186/s12929-023-00942-2 37380989 PMC10304357

[B70] Kwong B. Gai S. A. Elkhader J. Wittrup K. D. Irvine D. J. (2013). Localized immunotherapy via liposome-anchored Anti-CD137 + IL-2 prevents lethal toxicity and elicits local and systemic antitumor immunity. Cancer Res. 73, 1547–1558. 10.1158/0008-5472.CAN-12-3343 23436794 PMC3594475

[B71] Lakshmanan V. K. Jindal S. Packirisamy G. Ojha S. Lian S. Kaushik A. (2021). Nanomedicine-based cancer immunotherapy: recent trends and future perspectives. Cancer Gene Ther. 28, 911–923. 10.1038/s41417-021-00299-4 33558704

[B72] Larson R. C. Maus M. V. (2021). Recent advances and discoveries in the mechanisms and functions of CAR T cells. Nat. Rev. Cancer 21, 145–161. 10.1038/s41568-020-00323-z 33483715 PMC8353572

[B73] Lee J. W. Choi J. Choi Y. Kim K. Yang Y. Kim S. H. (2022). Molecularly engineered siRNA conjugates for tumor-targeted RNAi therapy. J. Control Release 351, 713–726. 10.1016/j.jconrel.2022.09.040 36152808

[B74] Li Q. Shi Z. Zhang F. Zeng W. Zhu D. Mei L. (2022). Symphony of nanomaterials and immunotherapy based on the cancer-immunity cycle. Acta Pharm. Sin. B 12, 107–134. 10.1016/j.apsb.2021.05.031 35127375 PMC8799879

[B75] Li X. Guo X. Ling J. Tang Z. Huang G. He L. (2021). Nanomedicine-based cancer immunotherapies developed by reprogramming tumor-associated macrophages. Nanoscale 13, 4705–4727. 10.1039/d0nr08050k 33625411

[B76] Li X. Yan X. Wang Y. Kaur B. Han H. Yu J. (2023). The notch signaling pathway: a potential target for cancer immunotherapy. J. Hematol. Oncol. 16, 45. 10.1186/s13045-023-01439-z 37131214 PMC10155406

[B77] Liang H. Deng L. Hou Y. Meng X. Huang X. Rao E. (2017). Host STING-dependent MDSC mobilization drives extrinsic radiation resistance. Nat. Commun. 8, 1736. 10.1038/s41467-017-01566-5 29170400 PMC5701019

[B78] Liu J. Ren L. Li S. Li W. Zheng X. Yang Y. (2021). The biology, function, and applications of exosomes in cancer. Acta Pharm. Sin. B 11, 2783–2797. 10.1016/j.apsb.2021.01.001 34589397 PMC8463268

[B79] Liu Y. Chang R. Xing R. Yan X. (2023). Bioactive peptide nanodrugs based on supramolecular assembly for boosting immunogenic cell death-induced cancer immunotherapy. Small Methods 7, e2201708. 10.1002/smtd.202201708 36720041

[B80] Liu Y. Qiao L. Zhang S. Wan G. Chen B. Zhou P. (2018). Dual pH-responsive multifunctional nanoparticles for targeted treatment of breast cancer by combining immunotherapy and chemotherapy. Acta Biomater. 66, 310–324. 10.1016/j.actbio.2017.11.010 29129789

[B81] Liu Y. T. Sun Z. J. (2021). Turning cold tumors into hot tumors by improving T-cell infiltration. Theranostics 11, 5365–5386. 10.7150/thno.58390 33859752 PMC8039952

[B82] Luo M. Wang H. Wang Z. Cai H. Lu Z. Li Y. (2017). A STING-activating nanovaccine for cancer immunotherapy. Nat. Nanotechnol. 12, 648–654. 10.1038/nnano.2017.52 28436963 PMC5500418

[B83] Maalej K. M. Merhi M. Inchakalody V. P. Mestiri S. Alam M. Maccalli C. (2023). CAR-cell therapy in the era of solid tumor treatment: current challenges and emerging therapeutic advances. Mol. Cancer 22, 20. 10.1186/s12943-023-01723-z 36717905 PMC9885707

[B84] Meltzer P. S. Helman L. J. (2021). New horizons in the treatment of osteosarcoma. N. Engl. J. Med. 385, 2066–2076. 10.1056/NEJMra2103423 34818481

[B85] Mi Y. Smith C. C. Yang F. Qi Y. Roche K. C. Serody J. S. (2018). A dual immunotherapy nanoparticle improves T-cell activation and cancer immunotherapy. Adv. Mater 30, e1706098. 10.1002/adma.201706098 29691900 PMC6003883

[B86] Min Y. Roche K. C. Tian S. Eblan M. J. McKinnon K. P. Caster J. M. (2017). Antigen-capturing nanoparticles improve the abscopal effect and cancer immunotherapy. Nat. Nanotechnol. 12, 877–882. 10.1038/nnano.2017.113 28650437 PMC5587366

[B87] Mishchenko T. Mitroshina E. Balalaeva I. Krysko O. Vedunova M. Krysko D. V. (2019). An emerging role for nanomaterials in increasing immunogenicity of cancer cell death. Biochim. Biophys. Acta Rev. Cancer 1871, 99–108. 10.1016/j.bbcan.2018.11.004 30528646

[B88] Nasiri F. Kazemi M. Mirarefin S. M. J. Mahboubi Kancha M. Ahmadi Najafabadi M. Salem F. (2022). CAR-T cell therapy in triple-negative breast cancer: hunting the invisible devil. Front. Immunol. 13, 1018786. 10.3389/fimmu.2022.1018786 36483567 PMC9722775

[B89] Olivo Pimentel V. Marcus D. van der Wiel A. M. Lieuwes N. G. Biemans R. Lieverse R. I. (2021). Releasing the brakes of tumor immunity with anti-PD-L1 and pushing its accelerator with L19-IL2 cures poorly immunogenic tumors when combined with radiotherapy. J. Immunother. Cancer 9, e001764. 10.1136/jitc-2020-001764 33688020 PMC7944996

[B90] Ovais M. Guo M. Chen C. (2019). Tailoring nanomaterials for targeting tumor-associated macrophages. Adv. Mater 31, e1808303. 10.1002/adma.201808303 30883982

[B91] Pathria P. Louis T. L. Varner J. A. (2019). Targeting tumor-associated macrophages in cancer. Trends Immunol. 40, 310–327. 10.1016/j.it.2019.02.003 30890304

[B92] Porter D. L. Levine B. L. Kalos M. Bagg A. June C. H. (2011). Chimeric antigen receptor-modified T cells in chronic lymphoid leukemia. N. Engl. J. Med. 365, 725–733. 10.1056/NEJMoa1103849 21830940 PMC3387277

[B93] Postow M. A. Sidlow R. Hellmann M. D. (2018). Immune-related adverse events associated with immune checkpoint blockade. N. Engl. J. Med. 378, 158–168. 10.1056/NEJMra1703481 29320654

[B94] Qian Y. Qiao S. Dai Y. Xu G. Dai B. Lu L. (2017). Molecular-targeted immunotherapeutic strategy for melanoma via dual-targeting nanoparticles delivering small interfering RNA to tumor-associated macrophages. ACS Nano 11, 9536–9549. 10.1021/acsnano.7b05465 28858473

[B95] Quintin J. Saeed S. Martens J. H. A. Giamarellos-Bourboulis E. J. Ifrim D. C. Logie C. (2012). Candida albicans infection affords protection against reinfection via functional reprogramming of monocytes. Cell Host Microbe 12, 223–232. 10.1016/j.chom.2012.06.006 22901542 PMC3864037

[B96] Ramesh A. Brouillard A. Kulkarni A. (2021). Supramolecular nanotherapeutics for macrophage immunotherapy. ACS Appl. Bio Mater 4, 4653–4666. 10.1021/acsabm.1c00342 35007018

[B97] Ramesh A. Brouillard A. Kumar S. Nandi D. Kulkarni A. (2020). Dual inhibition of CSF1R and MAPK pathways using supramolecular nanoparticles enhances macrophage immunotherapy. Biomaterials 227, 119559. 10.1016/j.biomaterials.2019.119559 31670078 PMC7238715

[B98] Ramesh A. Malik V. Brouillard A. Kulkarni A. (2022). Supramolecular nanotherapeutics enable metabolic reprogramming of tumor-associated macrophages to inhibit tumor growth. J. Biomed. Mater Res. A 110, 1448–1459. 10.1002/jbm.a.37391 35388955

[B99] Rancoule C. Magné N. Vallard A. Guy J. B. Rodriguez-Lafrasse C. Deutsch E. (2016). Nanoparticles in radiation oncology: from bench-side to bedside. Cancer Lett. 375, 256–262. 10.1016/j.canlet.2016.03.011 26987625

[B100] Rao L. Wu L. Liu Z. Tian R. Yu G. Zhou Z. (2020). Hybrid cellular membrane nanovesicles amplify macrophage immune responses against cancer recurrence and metastasis. Nat. Commun. 11, 4909. 10.1038/s41467-020-18626-y 32999291 PMC7527506

[B101] Ren E. Wang Y. Liang T. Zheng H. Shi J. Cheng Z. (2023). Local drug delivery techniques for triggering immunogenic cell death. Small Methods 2023, e2300347. 10.1002/smtd.202300347 37259275

[B102] Revia R. A. Stephen Z. R. Zhang M. (2019). Theranostic nanoparticles for RNA-based cancer treatment. Acc. Chem. Res. 52, 1496–1506. 10.1021/acs.accounts.9b00101 31135134 PMC6701180

[B103] Ritter J. Bielack S. S. (2010). Osteosarcoma. Ann. Oncol. 21 (7), vii320–5. 10.1093/annonc/mdq276 20943636

[B104] Rothschilds A. M. Wittrup K. D. (2019). What, why, where, and when: bringing timing to immuno-oncology. Trends Immunol. 40, 12–21. 10.1016/j.it.2018.11.003 30545676

[B105] Sharabi A. B. Nirschl C. J. Kochel C. M. Nirschl T. R. Francica B. J. Velarde E. (2015). Stereotactic radiation therapy augments antigen-specific PD-1-mediated antitumor immune responses via cross-presentation of tumor antigen. Cancer Immunol. Res. 3, 345–355. 10.1158/2326-6066.CIR-14-0196 25527358 PMC4390444

[B106] Song Y. Tang C. Yin C. (2018). Combination antitumor immunotherapy with VEGF and PIGF siRNA via systemic delivery of multi-functionalized nanoparticles to tumor-associated macrophages and breast cancer cells. Biomaterials 185, 117–132. 10.1016/j.biomaterials.2018.09.017 30241030

[B107] Srivastava R. M. Trivedi S. Concha-Benavente F. Gibson S. P. Reeder C. Ferrone S. (2017). CD137 stimulation enhances cetuximab-induced natural killer: dendritic cell priming of antitumor T-cell immunity in patients with head and neck cancer. Clin. Cancer Res. 23, 707–716. 10.1158/1078-0432.CCR-16-0879 27496866 PMC5290200

[B108] Stagg J. Golden E. Wennerberg E. Demaria S. (2023). The interplay between the DNA damage response and ectonucleotidases modulates tumor response to therapy. Sci. Immunol. 8, eabq3015. 10.1126/sciimmunol.abq3015 37418547 PMC10394739

[B109] Tuettenberg A. Steinbrink K. Schuppan D. (2016). Myeloid cells as orchestrators of the tumor microenvironment: novel targets for nanoparticular cancer therapy. Nanomedicine (Lond) 11, 2735–2751. 10.2217/nnm-2016-0208 27658725

[B110] van der Meel R. Vehmeijer L. J. Kok R. J. Storm G. van Gaal E. V. (2013). Ligand-targeted particulate nanomedicines undergoing clinical evaluation: current status. Adv. Drug Deliv. Rev. 65, 1284–1298. 10.1016/j.addr.2013.08.012 24018362

[B111] Walle T. Martinez Monge R. Cerwenka A. Ajona D. Melero I. Lecanda F. (2018). Radiation effects on antitumor immune responses: current perspectives and challenges. Ther. Adv. Med. Oncol. 10, 1758834017742575. 10.1177/1758834017742575 29383033 PMC5784573

[B112] Wang S. W. Fite B. Z. Kare A. J. Wu B. Raie M. Tumbale S. K. (2022a). Multiomic analysis for optimization of combined focal and immunotherapy protocols in murine pancreatic cancer. Theranostics 12, 7884–7902. 10.7150/thno.73218 36451859 PMC9706583

[B113] Wang S. W. Gao C. Zheng Y. M. Yi L. Lu J. C. Huang X. Y. (2022b). Current applications and future perspective of CRISPR/Cas9 gene editing in cancer. Mol. Cancer 21, 57. 10.1186/s12943-022-01518-8 35189910 PMC8862238

[B114] Wang Y. Zhou K. Huang G. Hensley C. Huang X. Ma X. (2014). A nanoparticle-based strategy for the imaging of a broad range of tumours by nonlinear amplification of microenvironment signals. Nat. Mater 13, 204–212. 10.1038/nmat3819 24317187 PMC3946908

[B115] Wei S. C. Levine J. H. Cogdill A. P. Zhao Y. Anang N. A. S. Andrews M. C. (2017). Distinct cellular mechanisms underlie anti-CTLA-4 and anti-PD-1 checkpoint blockade. Cell 170, 1120–1133. 10.1016/j.cell.2017.07.024 28803728 PMC5591072

[B116] Wei X. Yang M. (2023). Cell- and subcellular organelle-targeting nanoparticle-mediated breast cancer therapy. Front. Pharmacol. 14, 1180794. 10.3389/fphar.2023.1180794 37089933 PMC10117787

[B117] Xia Y. Yang R. Wang H. Hou Y. Li Y. Zhu J. (2022). Biomaterials delivery strategies to repair spinal cord injury by modulating macrophage phenotypes. J. Tissue Eng. 13, 20417314221143059. 10.1177/20417314221143059 36600997 PMC9806413

[B118] Xin Y. Huang M. Guo W. W. Huang Q. Zhang L. Z. Jiang G. (2017). Nano-based delivery of RNAi in cancer therapy. Mol. Cancer 16, 134. 10.1186/s12943-017-0683-y 28754120 PMC5534073

[B119] Xu X. Liu C. Wang Y. Koivisto O. Zhou J. Shu Y. (2021). Nanotechnology-based delivery of CRISPR/Cas9 for cancer treatment. Adv. Drug Deliv. Rev. 176, 113891. 10.1016/j.addr.2021.113891 34324887

[B120] Yan W. Lang T. Qi X. Li Y. (2020). Engineering immunogenic cell death with nanosized drug delivery systems improving cancer immunotherapy. Curr. Opin. Biotechnol. 66, 36–43. 10.1016/j.copbio.2020.06.007 32673944

[B121] Yang M. Li J. Gu P. Fan X. (2021). The application of nanoparticles in cancer immunotherapy: targeting tumor microenvironment. Bioact. Mater 6, 1973–1987. 10.1016/j.bioactmat.2020.12.010 33426371 PMC7773537

[B122] Yin W. M. Li Y. W. Gu Y. Q. Luo M. (2020). Nanoengineered targeting strategy for cancer immunotherapy. Acta Pharmacol. Sin. 41, 902–910. 10.1038/s41401-020-0417-3 32398683 PMC7470800

[B123] Yu S. Xiao H. Ma L. Zhang J. Zhang J. (2023). Reinforcing the immunogenic cell death to enhance cancer immunotherapy efficacy. Biochim. Biophys. Acta Rev. Cancer 1878, 188946. 10.1016/j.bbcan.2023.188946 37385565

[B124] Yu S. Yi M. Qin S. Wu K. (2019). Next generation chimeric antigen receptor T cells: safety strategies to overcome toxicity. Mol. Cancer 18, 125. 10.1186/s12943-019-1057-4 31429760 PMC6701025

[B125] Yuan H. Jiang W. von Roemeling C. A. Qie Y. Liu X. Chen Y. (2017). Multivalent bi-specific nanobioconjugate engager for targeted cancer immunotherapy. Nat. Nanotechnol. 12, 763–769. 10.1038/nnano.2017.69 28459470

[B126] Zhang X. Bai X. C. Chen Z. J. (2020). Structures and mechanisms in the cGAS-STING innate immunity pathway. Immunity 53, 43–53. 10.1016/j.immuni.2020.05.013 32668227

[B127] Zhang Y. Li N. Suh H. Irvine D. J. (2018). Nanoparticle anchoring targets immune agonists to tumors enabling anti-cancer immunity without systemic toxicity. Nat. Commun. 9, 6. 10.1038/s41467-017-02251-3 29295974 PMC5750237

[B128] Zhao C. Pang X. Yang Z. Wang S. Deng H. Chen X. (2022). Nanomaterials targeting tumor associated macrophages for cancer immunotherapy. J. Control Release 341, 272–284. 10.1016/j.jconrel.2021.11.028 34813877

[B129] Zhao D. Huang X. Zhang Z. Ding J. Cui Y. Chen X. (2021). Engineered nanomedicines for tumor vasculature blockade therapy. Wiley Interdiscip. Rev. Nanomed Nanobiotechnol 13, e1691. 10.1002/wnan.1691 33480163

[B130] Zheng N. Liu W. Li B. Nie H. Liu J. Cheng Y. (2019). Co-delivery of sorafenib and metapristone encapsulated by CXCR4-targeted PLGA-PEG nanoparticles overcomes hepatocellular carcinoma resistance to sorafenib. J. Exp. Clin. Cancer Res. 38, 232. 10.1186/s13046-019-1216-x 31151472 PMC6544999

[B131] Zheng Y. Han Y. Sun Q. Li Z. (2022). Harnessing anti-tumor and tumor-tropism functions of macrophages via nanotechnology for tumor immunotherapy. Explor. (Beijing) 2, 20210166. 10.1002/EXP.20210166 PMC10190945 37323705

[B132] Zhou L. Zhang P. Wang H. Wang D. Li Y. (2020). Smart nanosized drug delivery systems inducing immunogenic cell death for combination with cancer immunotherapy. Acc. Chem. Res. 53, 1761–1772. 10.1021/acs.accounts.0c00254 32819102

[B133] Zhu J. Fan J. Xia Y. Wang H. Li Y. Feng Z. (2023). Potential therapeutic targets of macrophages in inhibiting immune damage and fibrotic processes in musculoskeletal diseases. Front. Immunol. 14, 1219487. 10.3389/fimmu.2023.1219487 37545490 PMC10400722

[B134] Zhu K. Y. Palli S. R. (2020). Mechanisms, applications, and challenges of insect RNA interference. Annu. Rev. Entomol. 65, 293–311. 10.1146/annurev-ento-011019-025224 31610134 PMC9939233

[B135] Zhu Y. Yu X. Thamphiwatana S. D. Zheng Y. Pang Z. (2020). Nanomedicines modulating tumor immunosuppressive cells to enhance cancer immunotherapy. Acta Pharm. Sin. B 10, 2054–2074. 10.1016/j.apsb.2020.08.010 33304779 PMC7714985

[B136] Zins K. Abraham D. (2020). Cancer immunotherapy: ttargeting tumor-associated macrophages by gene silencing. Methods Mol. Biol. 2115, 289–325. 10.1007/978-1-0716-0290-4_17 32006408

